# Primary hyperparathyroidism in an adolescent presenting with genu valgus progressing to extensive bone disease; a case report

**DOI:** 10.1186/s12902-023-01328-z

**Published:** 2023-03-31

**Authors:** Nipun Lakshitha de Silva, Mihiran Denagama Jayalath, W. K. Chaminda Sampath, Ranga Perera, Chandana Karunathilake

**Affiliations:** 1 Department of Clinical Sciences, Faculty of Medicine, General Sir John Kotelawala Defence University, Boralesgamuwa, Sri Lanka; 2 Medical Unit, University Hospital-General Sir John Kotelawala Defence University, Boralesgamuwa, Sri Lanka; 3 Department of Radiology, University Hospital-General Sir John Kotelawala Defence University, Boralesgamuwa, Sri Lanka

**Keywords:** Primary hyperparathyroidism, Genu valgus, Adolescent, Fracture

## Abstract

**Background:**

Primary hyperparathyroidism which is rare in adolescents presents commonly with non-specific symptoms and systemic complaints. Though there are few reported cases of genu valgus, genu valgus progressing to extensive bone disease despite mildly elevated calcium had not been reported before.

**Case presentation:**

A 12-year-old male had been evaluated for bilateral (left > right) genu valgus and short stature. Serum calcium and phosphate levels had been normal. X-ray of the femora and pelvic bones had not shown additional abnormalities. Valgus deformity progressed despite left femoral plating, and a left distal femoral medial closed wedge osteotomy had been performed at 15 years. Plain imaging at that time had shown localised osteopaenia. At the age of 17 years, he developed multiple fragility fractures of his left hip rendering him wheelchair-bound. Further evaluation revealed a serum PTH level of 2571 (10–65) pg/mL with calcium of 2.82 (2.2–2.6) mmol/L and inorganic phosphate of 1.7 (2.2–4.7) mg/dL. The lumbar spine DXA scan showed a Z-score of -5.8. A left parathyroid adenoma was localised and there was evidence of hyperparathyroid bone disease including brown tumours. He underwent left parathyroidectomy and left thyroid lobectomy after which his PTH level dropped to 4.03 pg/mL. He developed hypocalcaemia which was managed successfully with calcium and alfacalcidol replacement.

**Conclusions:**

Primary hyperparathyroidism can present with genu valgus in adolescents. Initial normocalcaemia which could be due to concomitant vitamin D deficiency could mask this leading to delayed diagnosis until severe irreversible bone disease ensues.

## Background

Primary hyperparathyroidism in adolescents is rare compared to that of adults [1]. While it is commonly recognised in adults with screening during the asymptomatic period, adolescents are commonly diagnosed when they are symptomatic [2, 3]. However, symptoms are commonly generalised and non-specific due to hypercalcaemia. These include bone pain, asthenia, weight loss, abdominal pain, vomiting and lethargy [1, 2]. In some parts of the world, bone disease including fractures and deformities as well as renal complications have been commonly reported even in younger patients [1, 4, 5].

Though bone disease had been described as a presenting feature of primary hyperparathyroidism in adolescents, it is extremely uncommon for it to present with genu valgus. In fact, primary hyperparathyroidism is not considered in the diagnostic workup of an adolescent with genu valgus [6]. There are several case reports of adolescents with primary hyperparathyroidism presenting with genu valgus. However, fractures have not been a common manifestation in them, and they were noted to have hypercalcaemia at the time of presentation. We report an adolescent who initially presented with genu valgus and normocalcaemia progressing to severe bone disease with multiple fractures despite having mild hypercalcaemia.

## Case presentation

An 18-year-old Sri Lankan male was referred for evaluation of short stature, multiple bone deformities and fragility fractures for six years.

**Fig. 1:**
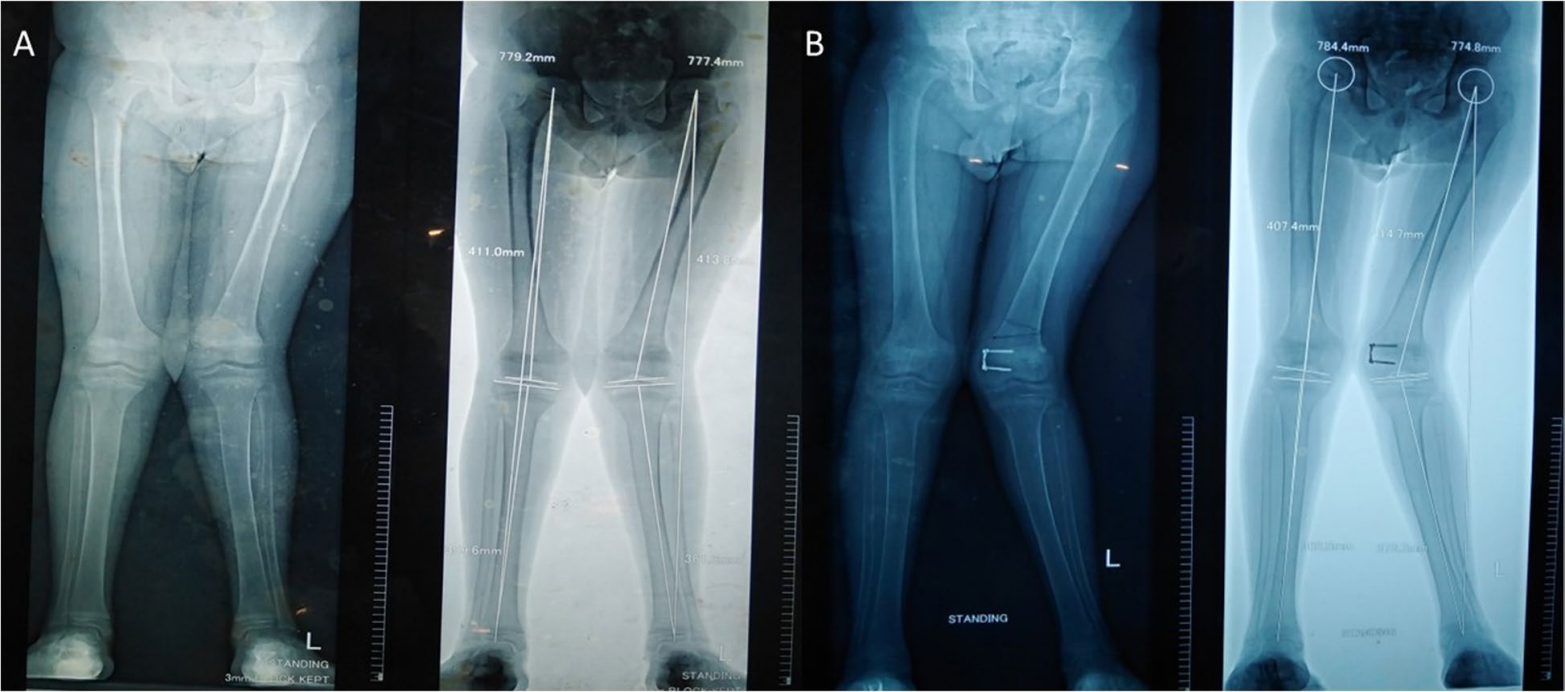
Standing X-rays of bilateral tibia/ femora and pelvic bones (AP view) at the presentation showing bilateral asymmetric genu valgus, but no reduction of bone density or abnormalities suggestive of rickets in epiphyseal plates (**A**). Follow-up x-ray of the same projection one year later demonstrating corrective left distal femoral osteotomy with internal fixation screws on the medial aspect of the distal femoral epiphysis and metaphysis. A more pronounced deformity is evident compared to the prior X-ray causing a mild pelvic tilt in the standing position (**B**)

He had been well until the age of 12 years with uncomplicated birth and childhood. At the age of 12 years, left and subsequently, right genu valgus and short stature were noted. He did not have any other problems during the initial presentation. The documented examination has not revealed any other abnormalities. Standing X-rays of bilateral tibia/ femora and pelvic bones (AP view) have shown bilateral asymmetric genu valgus, but no reduction of bone density or abnormalities suggestive of rickets in epiphyseal plates (Fig. 1 A).

Blood investigations at the initial presentation revealed an ionized calcium of 1.4 mmol/L (1.2–1.4 mmol/L) and the inorganic phosphate level was 1.1 mmol/L (0.95–1.75 mmol/L, age-based for 12–15 years). Serum creatinine, haemoglobin, alanine transaminase and aspartate transaminase were within the normal range. Serum vitamin D or parathyroid hormone levels had not been performed.

He underwent left femoral plating, but valgus deformity progressed over time. X-ray of the same projection done one year later demonstrated corrective left distal femoral osteotomy with internal fixation screws on the medial aspect of the distal femoral epiphysis and metaphysis. However, the deformity was more pronounced compared to the prior X-ray causing a mild pelvic tilt in the standing position (Fig. 1B).

**Fig. 2:**
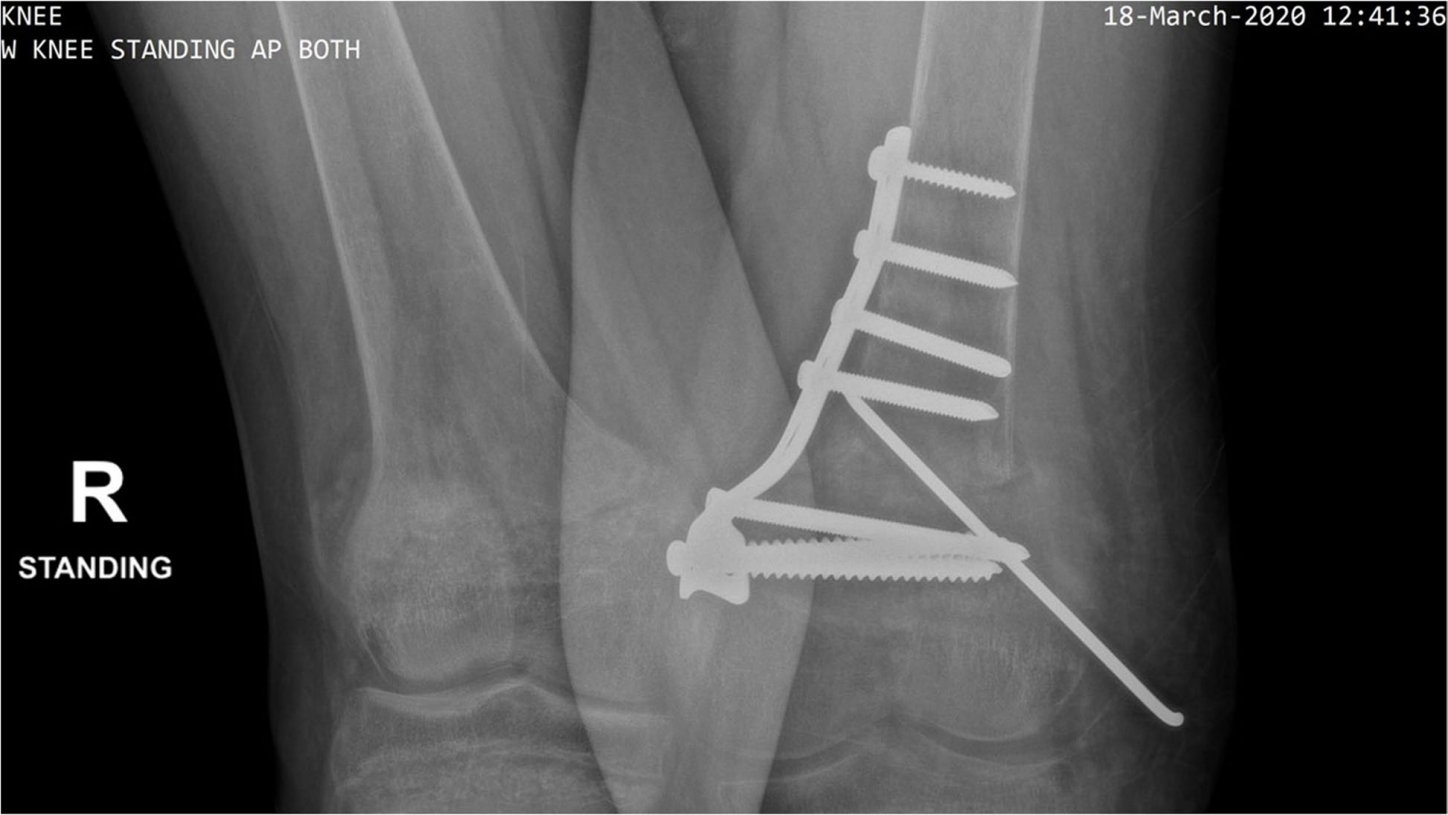
Post-operative x-ray of bilateral knee joints after the left distal femoral medial closed wedge osteotomy showing evidence of the second osteotomy with internal fixation screws in situ. Local osteopaenia is observed around the internal fixation screws. Some callus formation is seen laterally around the distal femoral metaphysis/epiphysis

Three years later, at the age of 15 years, he sought advice from another unit for the persistent genu valgus, where he underwent left distal femoral medial closed wedge osteotomy. Post-operative x-ray of the bilateral knee joints revealed evidence of the second osteotomy with internal fixation screws in situ. Local osteopaenia was noted around the internal fixation screws. Some callus formation is seen laterally around the distal femoral metaphysis/epiphysis (Fig. 2). He was prescribed vitamin D 1000 IU daily which was continued until the current presentation.

Two years later, at the age of 17 years, he developed a left femoral shaft fracture while standing up. Subsequent evaluation revealed multiple femoral fractures and he was unable to weight-bear since then. He was mobilised in a wheelchair since then. He has had normal puberty and intellectual development. He had been on a balanced non-vegetarian diet and there were no features of any other chronic illnesses. He was a product of non-consanguineous parents. Family history did not reveal any skeletal diseases or tumour syndromes.

His height was 147.5 cm. There was kyphosis and genu valgus. The examination was otherwise unremarkable.

**Fig. 3:**
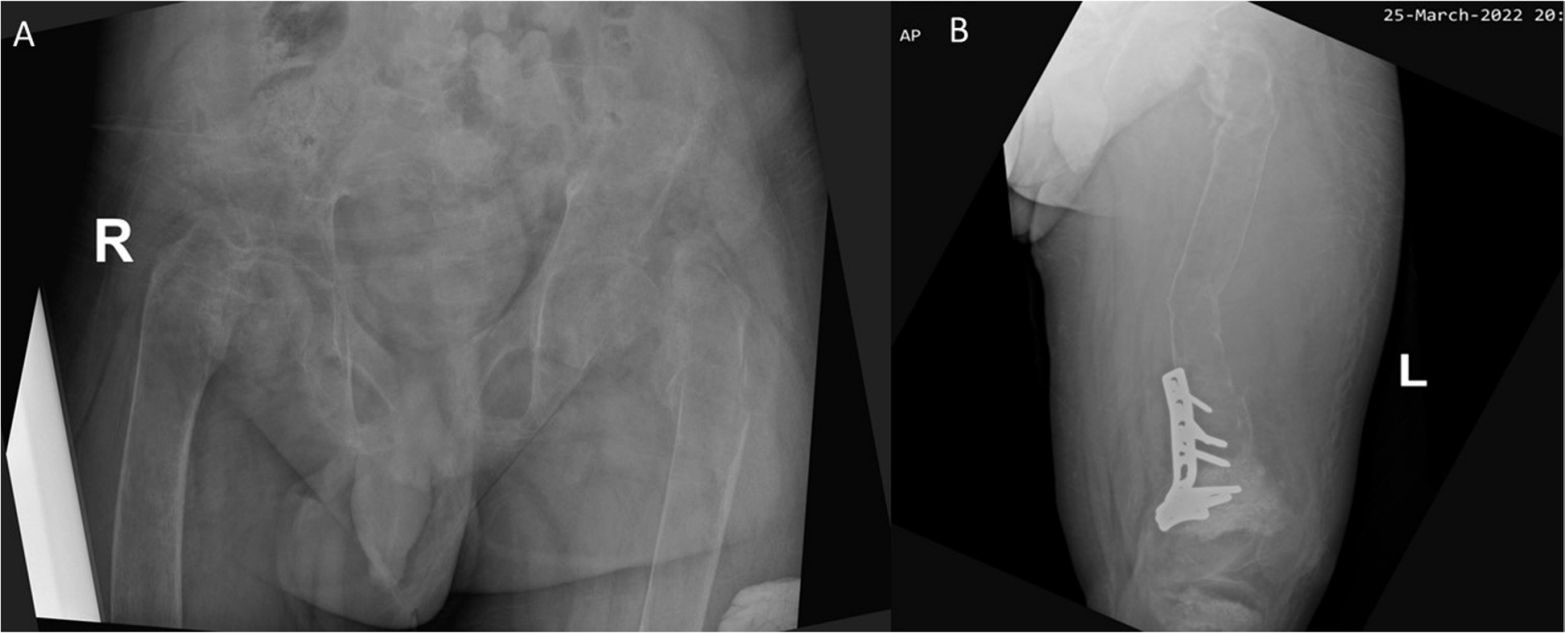
Generalised osteopenia and bilateral femoral neck fractures were noted in the x-ray of bilateral hip joints (**A**). Left femur x-ray showed marked osteopaenia, diffuse ground glass density in the medullary cavity of the femur and pathological fractures with marked deformity (**B**)

**Fig. 4:**
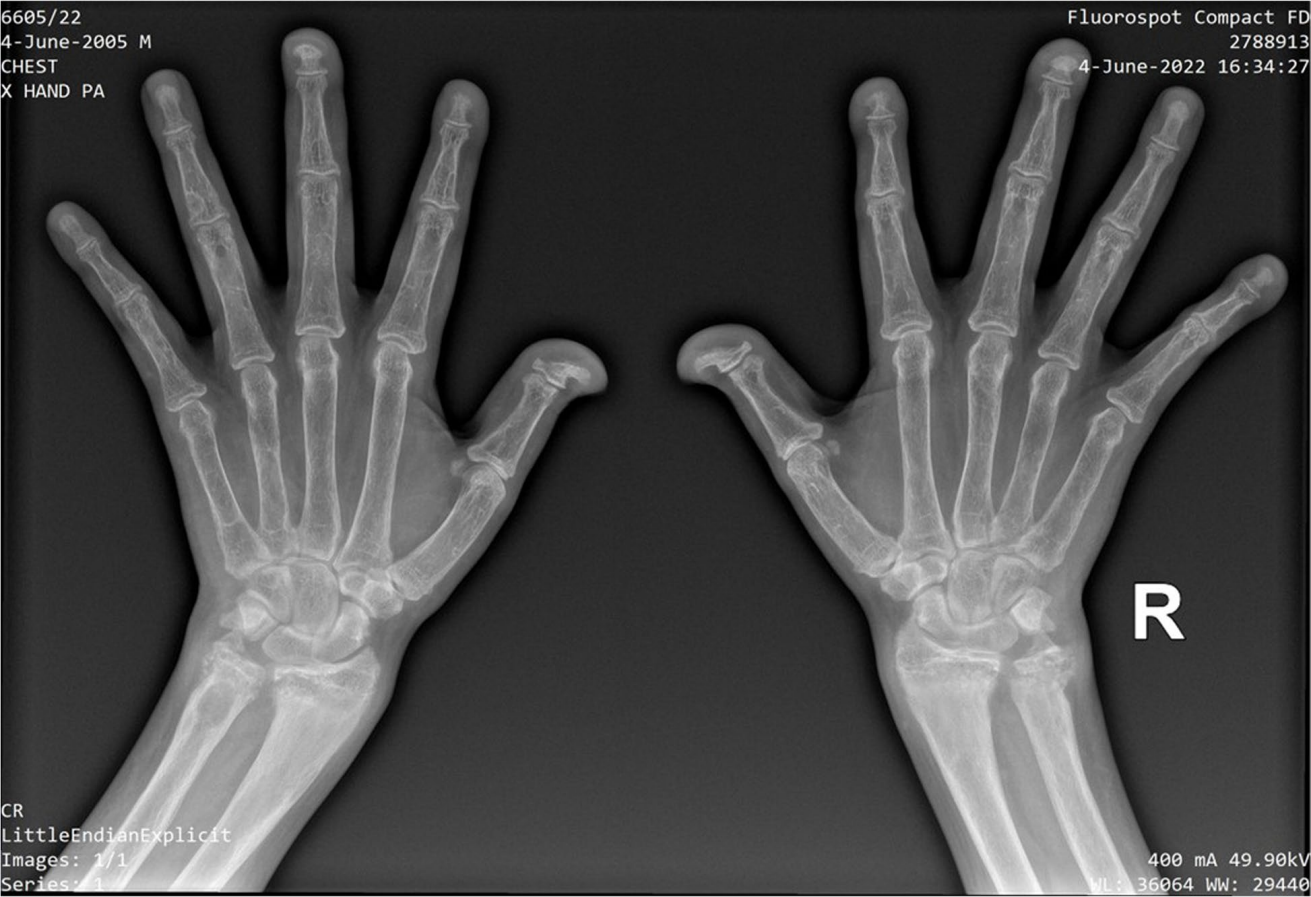
X-rays of the hands showed fully fused epiphyses, terminal tufting more marked in bilateral 2^nd^ and 3^rd^ fingers and osteopenia in the middle and distal phalanges of all the fingers as well as the distal aspect of the proximal phalanges bilaterally. There was no definite subperiosteal resorption. Early Madelung deformity was seen but, the length of the metacarpals was normal. There was no evidence of rickets

Generalised osteopenia and bilateral femoral neck fractures were noted in the x-ray of bilateral hip joints (Fig. 3A). Left femur x-ray showed marked osteopaenia, diffuse ground glass density in the medullary cavity of the femur and pathological fractures with marked deformities (Fig. 3B). X-rays of the hands showed fully fused epiphyses, terminal tufting more marked in bilateral 2^nd^ and 3^rd^ fingers and osteopenia in the middle and distal phalanges of all the fingers as well as the distal aspect of the proximal phalanges bilaterally. There was no definite subperiosteal resorption. Early Madelung deformity was seen but, the length of the metacarpals was normal. There was no evidence of rickets (Fig. 4). Laboratory investigations during this presentation are summarised in Table 1.

**Table 1:** Summary of blood investigations at the time of endocrine evaluation

Measurement	Results	Reference range
Haemoglobin (g/dL)	15.6	13.2–16.6
Creatinine (mg/dL)	0.74	0.7–1.3
ALT (IU/L)	27	< 36
AST (IU/L)	36	< 36
Albumin (g/L)	34.6	35–50
Alkaline phosphatase (IU/L)	4785	98–258
Total calcium (mmol/L)	2.82	2.2–2.6
Inorganic phosphate (mg/dL)	1.7	2.2–4.7
25-hydroxyvitamin D (ng/mL)	24.96	30–100
Intact PTH (pg/mL)	2571	10–65
Urine calcium creatinine ratio	0.27	< 0.2
TSH (mIU/L)	0.91	0.4–4

**Fig. 5:**
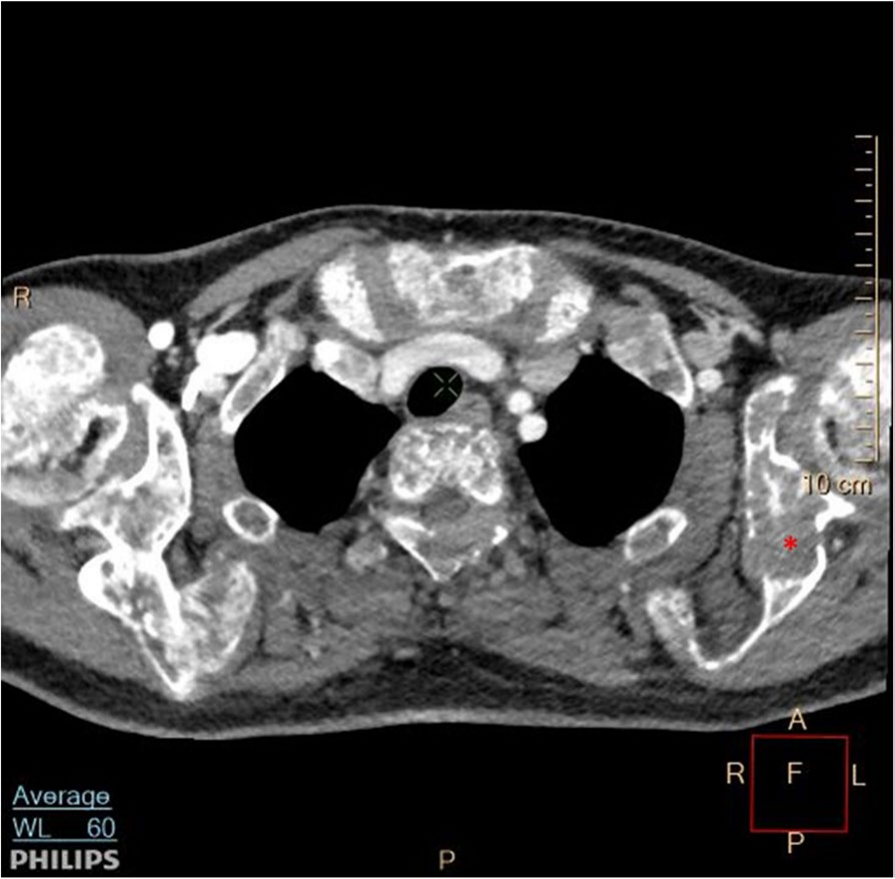
Contrast-enhanced CT scan of the neck and upper chest showing lytic lesions on scapulae suggestive of brown tumours

Laboratory investigations were in favour of primary hyperparathyroidism. The ultrasound scan of the neck showed a large well-defined heterogeneous hyperechoic lesion in the lower and mid portion of L/thyroid measuring 51 × 30 × 16 mm. Contrast-enhanced CT of the neck confirmed a well-defined rounded soft tissue density with moderate heterogeneous enhancement along the posterior aspect of the L/lobe of the thyroid. This was suggestive of a parathyroid adenoma. Additionally, diffusely scattered lytic bone lesions with associated bone expansion were noted in the imaged axial skeleton and upper humeri suggestive of brown tumours of hyperparathyroid bone disease (Fig. 5). The PTH level from the aspirate of guided FNAC was > 3000 pg/mL further confirming localisation.

DXA scan of the lumbar spine suggested very low bone density with a BMD of 0.122 g/cm^2^ and a Z-score of -5.8. There were no calculi or nephrocalcinosis in the ultrasound scan kidney-ureter-bladder.

He underwent left parathyroidectomy and left thyroid lobectomy since there was a concern about possible parathyroid malignancy peri-operatively. A nodular, capsulated and brownish parathyroid measuring 60 × 30 × 24 mm was removed. Histology confirmed a well-circumscribed parathyroid adenoma with a thin fibrous capsule. There were no bizarre cells or increased mitoses. One week after the surgery, the PTH level dropped to 4.03 pg/mL. He was discharged home on calcium carbonate containing elemental calcium of 1000 mg three times daily and alfacalcidol one microgram twice daily. Two months after the surgery, his alkaline phosphatase was 1811 IU/L and calcium and phosphate were within the normal range. His replacement doses were gradually decreased to calcium carbonate containing elemental calcium of 500 mg twice daily and alfacalcidol 0.5 µg twice daily six months after surgery. Currently, he is feeling well and has been followed up with a plan for future orthopaedic interventions. To date, he is mobilised with the help of a wheelchair.

The timeline of events is summarised in Table 2.

**Table 2:** Timeline of events with diagnostic tests and interventions

Month/year (age)	Events	Diagnostic tests	Interventions
February/2017 (12 years, 7 months)	Left > right genu valgus	X-ray- no changes of rickets or oteopaenia Normal calcium and phosphate	Left femoral plating
June/ 2018 (13 years, 11 months)	Worsening deformity	X-ray- progression of the deformity with a pelvic tilt	None
March/2020 (15 years, 8 months)	Presenting to a second orthopaedic unit with worsening deformity	X-ray- localised osteopaenia	Left distal femoral medial closed wedge osteotomy
November/ 2021 (17 years, 4 months)	Fragility fracture	X-ray- multiple fractures and severe osteopaenia	
July/ 2022 (18 years)	Endocrine evaluation	High serum PTH Parathyroid adenoma Hyperparathyroid bone disease	Left parathyroidectomy and left thyroid lobectomy

## Discussion and conclusions

Our patient initially presented with bilateral genu valgus and short stature with no other obvious abnormalities in physical examination and biochemistry. Subsequently, it progressed to severe disease with very low bone density and multiple fragility fractures. Yet, the calcium level was only mildly elevated. This course of the illness has not been reported before.

We performed a systematic literature review to identify reports of patients with primary hyperparathyroidism presenting with genu valgus. The search was done in PubMed on 1^st^ September 2022 with the search strategy as follows; (hyperparathyroidism[Title/Abstract]) AND (genu valg*[Title/Abstract] OR knock knee[Title/Abstract]). This provided 21 results. A similar search in Google Scholar obtained 9 results. After removing the duplicates, 27 abstracts were reviewed. Nineteen abstracts were selected to review full-text from which four were removed (English full-text not available- 3, duplicate—1) and fifteen were included. With eight additional records identified through cross-referencing, a total of 23 reports were included in the literature review [7–29] (Table 3).

**Table 3:** Summary of reported patients with hyperparathyroidism and genu valgus

Author, year	Age at presentation, sex	Age at diagnosis	Clinical features	Radiology	Total Calcium	Phosphate	ALP (IU/L)	PTH	Other
Dikova, 2021 [7]	9, F	12	B/L genu valgus, inability to walk, obesity	Fibrocystic osteodystrophy, cysts, brown tumours	3.73 mmol/L	0.8 mmol/L	4700	2238 pg/mL	Vit D- 14 nmol/L, Urine Ca- 6.16 mmol/L
	15, F	15	L/ genu valgus, waddling gait	Osteoporosis and bone cysts	3.15 mmol/L	0.87 mmol/L	1973	709 pg/mL	Vit D- 23.3 nmol/L
Lee, 2021	13, M	15	B/L genu valgus, short stature	No features typical of rickets	2.97 mmol/L	1.03 mmol/L	1354	154 pmol/L	
Yanrismet, 2019 [9]	13, M	13	B/L genu valgus, muscle weakness, difficulty in walking, bone and joint pain	Low bone density, epiphyseal widening, rugger-jersey spine, B/L SUFE	High	Low	NR	1301 pg/mL	Low vitamin D Low BMD
Rao, 2019 [10]	11, F	12	B/L genu valgus	Generalised osteopaenia and subchondral resorption	8.1 mg/dL	2.8 mg/dL	6690	2616 pg/mL	Vitamin D: 17.79 ng/mL
Paruk, 2019 [11]	14, M	17	B/L genu valgus, short stature, rickets features, delayed puberty	Features of rickets, B/L fractures of the metaphyseal region of the femur	3.02 mmol/L (corrected)	1.44 mmol/L	929	134.6 pmol/L (67.9 at the initial presentation)	BMD Z scores: -5 at hip and -4.9 at spine 24-h urinary calcium: 6.1 mmol 1,25 Vitamin D: 1384 pmol/L (48–108) at the initial presentation
	13, M	13	L/ genu valgus and r/ genu varus, quadriceps weakness and wasting	Features of rickets, Brown tumours, subperiosteal resorption, pepper pot skull	3.4 mmol/L (corrected)	0.97 mmol/L	1193	131.1 pmol/L	24-h urinary calcium: 7.5 mmol BMD Z core -2.4 at hip and -4.8 at spine
Khan, 2019 [12]	11, M	17	Muscle wasting, tender ribs, B/L genu valgus, eversion of ankle joints, flat feet short stature	flaring, widening and splaying of the distal femur and tibia, cupping and fraying without epiphyseal closure	16.3 mg/dL (7.9 mg/dL at the initial presentation)	3.1 mg/dL	1118 (867 at the initial presentation)	122.3 pg/mL	Vitamin D: 69.3 ng/mL
George, 2019 [13]	14, M	15	B/L genu valgus, rachitic rosary, Harrison sulcus, proximal myopathy	B/L SUFE	17.2 mg/dL	2.8 mg/dL	NR	1052 pg/mL	
Pradhan, 2018 [14]	NR, F	17	B/L genu valgus, wrist widening	Rickets features on hands, B/L SUFE	14.2 mg/dL	3.4 mg/dL	1400	1967 pg/mL	Vitamin D: 25.1 nmol/L, 24-h urinary calcium: 282 mg
	12, F	15	B/L genu valgus, hip dislocation, wrist widening, rachitic rosary and kyphosis, left hand and clavicle fractures	Generalised osteopaenia, increased trabecular pattern	High	NR	High	1250 pg/mL	Vitamin D: low
Arambewela, 2017 [15]	11, F	12	B/L genu valgus	B/L SUFE, rugger jersey spine, epiphyseal displacement of humeri, subperiosteal bone resorption, no rickets changes	1.5 mmol/L (ionised)	2.3 mg/dL	3210	796 pg/mL	Urinary calcium creatinine ratio: 0.06 Z score: -3.4 Vitamin D: 25 nmol/L
Zil, 2016 [16]	12, F	14	Short stature, B/L genu valgus, flat feet, pectus carinatum, scoliosis	Generalised osteopaenia, lytic and sclerotic lesions, brown tumours, no rickets changes	13.5 mg/dL	2 mg/dL	415	3203 pg/mL	Vitamin D: 24.9 ng/mL
Sharma, 2016 [17]	11, F	15	Genu valgus, pectus carinatum, proximal weakness	Generalised osteopaenia, subperiosteal resorption, cysts	13.2 mg/dL	2.64 mg/dL	10,280	> 2500 pg/mL	24-h urinary calcium: 300 mg Vitamin D: 18 ng/mL
Ganie, 2016 [29]	14, M	14	B/L genu valgus, features of rickets	Features of rickets	15.2 mg/dL	2.4 mg/dL	3936	1164 pg/mL	24-h urinary calcium: 460 mg Vitamin D: 18 ng/mL
	14, M	14	B/L genu valgus, features of rickets	Features of rickets and a lytic lesion in a phalanx	14.8 mg/dL	1.3 mg/dL	1652	657 pg/mL	24-h urinary calcium: 200 mg Vitamin D: 40 ng/mL
	15, M	15	Genu valgus, features of rickets and tibial shaft fracture	Features of rickets and tibial fractures	12 mg/dL	2.5 mg/dL	240	60 pg/mL	24-h urinary calcium: 350 mg Vitamin D: 19 ng/mL
Ramkumar, 2014 [18]	12, M	16	B/L genu valgus, arthralgia, polyuria	Brown tumour	11 mg/dL	3.7 mg/dL	2416	760 pg/mL	24-h urinary calcium: 570 mg Vitamin D: 9 ng/mL
	13, M	13	B/L genu valgus, abdominal pain, vomiting, myalgia	Brown tumours	10.7 mg/dL	3.7 mg/dL	1001	1136 pg/mL	24-h urinary calcium: 520 mg Vitamin D: 5.1 ng/mL
Ratnasingam, 2013 [19]	NR, F	16	B/L genu valgus	Generalised osteopaenia, subperiosteal resorption, terminal resorption	12.4 mg/dL	2.8 mg/dL	1136	1649 pg/mL	Vitamin D: 28 ng/mL 24-h urinary calcium: 196 mg
Dutta, 2013 [20]	8, F	12	Short stature, B/L genu valgus, ankle eversion deformity, flat feet	Generalised osteopaenia, widening of distal ends of long bones with splaying, cupping and fraying	7.8 mg/dL (After vitamin D correction: 11.9)	1.4 mg/dL	1322 (After vitamin D correction: 732)	811 pg/mL (After vitamin D correction: 632)	Vitamin D: 8.7 ng/mL (After vitamin D correction: 41)
Walczyk, 2011 [21]	NR, M	15	B/L genu valgus, overweight, hypertension, recurrent infections, seizures	osteoporosis	14.6 mg/dL	1.42 mg/dL	NR	880 pg/mL	Z score -1.15
Harman, 1999 [22]	14, F	11	B/L genu valgus	Lytic lesions in metacarpal bones	NR	NR	NR	NR	Extracted from a case series
Menon, 1994 [23]	8, F	14	B/L genu valgus, short stature, frontal bossing, rachitic rosary, lumbar lordosis, fixed adduction deformity of left leg	Generalised osteopaenia, subperiosteal resorptions, salt and pepper appearance on skull, erosions on lateral ends of clavicles, brown tumours	2.69 mmol/L	1.23 mmol/L	824	790 ng/L	24-h urinary calcium: 2.87 mmol
Kauffmann, 1993 [24]	13, F	14	B/L genu valgus, backache	Subperiosteal resorption of bone, skull demineralisation, B/L coxa vara	3.8 mmol/L	0.76 mmol/L	6612	1066 pg/mL	Urinary excretion of calcium: 26 mg/g/day 1,25 OH D3: 125 ng/mL (20–80)
Rapaport, 1986 [25]	12, F	15	B/L genu valgus, weight loss, polyuria, calculi, GI and neuropsychiatric	‘Moth-eaten’ skull	17.1 mg/dL	1.6 mg/dL	NR	NR	
	13, M	15	B/L genu valgus, calculi	Osteoporosis	13.7 mg/dL	1.5 mg/dL	NR	NR	
Lloyd, 1965 [26]	12, M	14	B/L genu valgus, weakness, neurological manifestations, palpable beading in the ribs, thoracic scoliosis	Subperiosteal erosions, cyst like lesions, enlarged, irregular epiphyseal plates	17.6 mg/dL	3.1 mg/dL	52 King-Amstrong units	NR	24-h urinary calcium: 457 mg
Balch, 1953 [27]	17, F	21	B/L genu valgus, vomiting, nocturia, tenderness over ribs, clubbing	Generalised demineralisation, cystic areas	21.2 mg/dL	2 mg/dL	127 King-Amstrong units	NR	
McCulre, 1945 [28]	14, F	14	B/L genu valgus	Low bone density with coarse trabeculations,	15.3 mg/dL	2.6 mg/dL	6.37 Bodansky units	NR	Urine calcium excretion six times elevated

*ALP* alkaline phosphatase, *B/L* bilateral, *BMD* bone mineral density, *L* left, *NR* not reported, *PTH* parathyroid hormone, *R* right, *SUFE* slipped upper femoral epiphyses

Thirty patients have been reported (16 females). The median age at presentation was 13 years (Age at diagnosis taken as age at presentation for three patients). One patient had low serum calcium levels [10], whereas all the other patients were hypercalcaemic. All patients with reported histology have had parathyroid adenomas. In addition to genu valgus, 12 had features of rickets clinically or radiologically. Osteopaenia was commonly observed in radiological studies. Sixteen patients were reported to have radiological features characteristic of hyperparathyroid bone disease including cysts, brown tumours and subperiosteal resorption. Slipped upper femoral epiphyses were also noted in some.

Fractures have been observed only in two patients. One was a 17-year-old boy who initially presented at 14 years of age with lower limb pain and bilateral genu valgus [11]. At the diagnosis, his corrected calcium was 3.02 mmol/L. DXA scan showed BMD Z scores of -5 at the hip and -4.9 at the spine. He was found to have previously unrecognised bilateral femur shaft fractures which have partially healed. The other patient was a 15-year-old male who presented with bone pains and a right tibial shaft fracture [29]. However, this boy had been treated for right tibial osteomyelitis which also might have contributed to this complication. His calcium was 12 mg/dL at the time of diagnosis. It was notable both these boys had features of rickets, but not hyperparathyroid bone disease.

Our patient’s clinical picture is distinct from all the patients reported above since he initially presented with bilateral genu valgus and then it progressed to severe bone disease with very low bone density and fragility fractures. He was found to have features of hyperparathyroid bone disease, but no definite evidence of rickets. Though his PTH level was grossly elevated, there was only mild hypercalcaemia. In fact, his calcium at the initial presentation was normal. Vitamin D deficiency could have masked hypercalcemia at the initial presentation. However, his vitamin D level was 24.96 ng/mL at the time of endocrine evaluation which was only slightly below the reference range. Calcium: phosphorous ratio could have been a useful tool at the initial evaluation since his calcium was at the upper end of the reference range and phosphate was at the lower half of the reference range for his age. This index has emerged as a reliable tool for identifying patients with primary hyperparathyroidism [30]. Age-specific reference ranges are needed in interpreting phosphate levels and calcium: phosphate ratio. The presence of osteopaenia in subsequent plain radiographs would have been another clue to an alternative diagnosis.

The mechanism of genu valgus in primary hyperparathyroidism is not well established. However, one plausible mechanism is the direct effect of increased parathyroid hormone levels on the growth plate during puberty [18]. It could be partly due to the concomitant vitamin D deficiency. Notably, most reported cases are from South Asian populations where vitamin D deficiency is more prevalent. When undiagnosed primary hyperparathyroidism continues as in our patient and the patient reported by Paruk et al., severe bone disease establishes with reduced bone mineral density and fractures. Severe hyperparathyroid bone disease was also evident in our patient further confirming the severe skeletal involvement of the disease.

This case report highlights the importance of close surveillance for underlying metabolic diseases in patients presenting with genu valgus. Though the initial calcium was normal, a high index of suspicion is warranted particularly in the setting of possible concomitant vitamin D deficiency and progressing disease with osteopaenia in the plain radiograph.

## Data Availability

Not applicable.
